# Do community pharmacists have the attitudes and knowledge to support evidence based self-management of low back pain?

**DOI:** 10.1186/1471-2474-8-10

**Published:** 2007-01-31

**Authors:** Jonathan Silcock, Jennifer Klaber Moffett, Hilary Edmondson, Gordon Waddell, A Kim Burton

**Affiliations:** 1 School of Healthcare, University of Leeds, PO Box 214, Leeds, LS2 9UT, UK; 2 Institute of Rehabilitation, University of Hull, 215 Anlaby Road, Hull, HU3 2PG, UK; 3 Hull and East Riding Pharmacy Research Network, Room SC48 Trust Headquarters, Willerby Hill, Willerby, HU10 6ED, UK; 4 UnumProvident Centre for Psychosocial and Disability Research, 51a Park Place, Cardiff University, Cardiff, CF10 3AT, UK; 5 Spinal Research Unit, University of Huddersfield, 30 Queen Street, Huddersfield, West Yorks, HD1 2SP, UK

## Abstract

**Background:**

In many countries, community pharmacists can be consulted without appointment in a large number of convenient locations. They are in an ideal position to give advice to patients at the onset of low back pain and also reinforce advice given by other healthcare professionals. There is little specific information about the quality of care provided in the pharmacy for people with back pain. The main objectives of this survey were to determine the attitudes, knowledge and reported practice of English pharmacists advising people who present with acute or chronic low back pain.

**Methods:**

A questionnaire was designed for anonymous self-completion by pharmacists attending continuing education sessions. Demographic questions were designed to allow comparison with a national pharmacy workforce survey. Attitudes were measured with the *Back Beliefs Questionnaire *(BBQ) and questions based on the *Working Backs Scotland *campaign. Questions about the treatment of back pain in the community pharmacy were written (or adapted) to reflect and characterise the nature of practice. In response to two clinical vignettes, respondents were asked to select proposals that they would recommend in practice.

**Results:**

335 responses from community pharmacists were analysed. Middle aged pharmacists, women, pharmacy managers and locums were over-represented compared to registration and workforce data. The mean (SD) BBQ score for the pharmacists was 31.37 (5.75), which was slightly more positive than in similar surveys of other groups. Those who had suffered from back pain seem to demonstrate more confidence (fewer negative feelings, more advice opportunities and better advice provision) in their perception of advice given in the pharmacy. Awareness of written information that could help to support practice was low. Reponses to the clinical vignettes were generally in line with the evidence base. Pharmacists expressed some caution about recommending activity. Most respondents said they would benefit from more education about back pain.

**Conclusion:**

Those sampled generally expressed positive attitudes about back pain and were able to offer evidence based advice. Pharmacists may benefit from training to increase their ability and confidence to offer support for self-care in back pain. Further research would be useful to clarify the representativeness of the sample.

## Background

Back pain is the most commonly reported physical symptom, after headache and tiredness [1]. It results in a great deal of disability and distress especially in industrialised countries. Attitudes and beliefs about its course and management play an important role: they impact on the severity and extent of disability and distress reported. Evidence based treatment for non-specific low back pain includes brief educational interventions, short term painkillers, supervised exercise and counselling [2]. To maximise the chances of successful treatment and minimise variation in care, it is important that all healthcare professionals understand the basic principles of back pain management. They also need to be confident that an active management strategy is the best approach as recommended in international guidelines [3]. Family physicians (known in the UK as general medical practitioners: GPs) are often the first port of call for people with back pain, but community pharmacists and nurses are important sources of advice for many people.

Community pharmacists can be consulted without appointment in a large number of convenient locations. They are in an ideal position to give timely advice to patients at the onset of low back pain and also reinforce advice given by other healthcare professionals. For example they can:

• provide over-the-counter (OTC) analgesics and non-steroidal anti-inflammatory drugs (NSAIDs), when it is safe to do so

• reassure and provide appropriate information and/or advice

• advise those with "red flags" to seek medical care

In fact, community pharmacists and their staff do all these things, but there is little specific information about the quality of care provided in the pharmacy for people with back pain. The quality of this care will be determined by factors that are professional, patient related and environmental. The main professional factors are the knowledge, skills and attitudes of individual practitioners. Most importantly, it would be helpful to be sure that bed rest is not being recommended, since this is considered harmful in most cases. The attitudes and practice of specialist physicians, family physicians and physical therapists have been previously investigated [4-7].

Campaigns to make patients and professionals more aware of current guidelines have taken place in the UK and Australia. In Australia, a large public health campaign was successful in improving attitudes to back pain. The campaign also reduced functional disability related to back pain in the general population and the economic impact of back pain [8]. In Scotland, there has been a campaign to try to bring about change in the attitudes lay public and health professionals have about back pain and how to cope with it. Population surveys show that this campaign achieved 60% penetration and a 20% positive shift in public attitudes towards keeping active [9].

In England, a new National Health Service (NHS) contract for community pharmacists was implemented in April 2005. This gives them an expanded role in which they are expected to take a more central position managing patients with long term conditions and supporting self-care [10]. It is therefore timely to carry out a survey to determine how prepared pharmacists are to take up this role in relation to back pain. Additionally, information from a survey can be used to design appropriate educational resources if required for pharmacists, their staff and people with back pain. The survey objectives were to:

• determine the attitudes, knowledge and reported practice of community pharmacists advising people who present with acute or chronic low back pain

• compare current reported practice with evidence-based clinical guidelines

• make recommendations about the pharmacists' role and training needs in relation to self-management of back pain

## Methods

### Questionnaire design

A questionnaire was designed for anonymous self-completion and organised in five sections:

• demographic questions about the respondents

• attitudes towards back pain and its treatment

• frequency and quality of back pain advice in the pharmacy

• clinical case studies (2 vignettes)

• education and training needs

Demographic and potential explanatory variables collected were: year of first professional registration, age, gender, personal experience of back pain (none, short term, long standing), job role and hours of work. Responses to demographic questions were designed to allow comparison with a national pharmacy workforce survey (Table 1) [11].

Attitudes were measured with (a) the *Back Beliefs Questionnaire *(BBQ), which is a measure of beliefs about the inevitable consequences of back pain; the range of possible scores is 5–45 where high scores represent positive beliefs about long term outcomes [12] (b) questions mainly based on *Working Backs Scotland *(WBS). Two questions were taken directly from the WBS campaign; one question about returning to work was adapted from Buchbinder [13]; and a new question about using painkillers was written (Table 2). Participants were invited to respond to these questions on a 5-point Likert scale similar to that employed by the BBQ.

Questions about treatment of back pain in the community pharmacy are summarised in Table 3; responses were invited on a binary scale: agree/disagree. The first of these questions was based on a similar question used in GP questionnaires by both Buchbinder and Chaudhary [13,14]. The rest of these questions were written to reflect and help to characterise the nature of community pharmacy practice. A further question about recommending *The Back Book *[15] was adapted from Chaudhary [14] and read "I recommend *The Back Book *to people with back pain"; in an additional response pharmacists could say that they were unfamiliar with the publication.

Vignettes were designed by consultation between the authors to simulate practice situations and assess pharmacists' knowledge of the evidence base. Vignette A was a straightforward case of acute back pain with a good prognosis:

"A woman comes into your pharmacy one lunchtime. She wants to buy the strongest painkillers she can get without a prescription. You find out that she works in the local supermarket and is 35 years old; the pain is in her low back and bottom. Otherwise she is well and her only regular medication is 'the pill'. Her back went last week when stacking shelves and she's been off work since. She's doing as little as possible so her back doesn't go again, but even now it's hard to get up in the morning and sort the children out. The only time she can properly rest is in the evening. She thinks going back to work will be really hard and wants some help."

Vignette B was a more complicated chronic case associated with long term disability and accompanying distress:

"A man comes into your pharmacy near closing time one afternoon. He would have called in earlier, but he's been having trouble getting going in the morning and needs to rest after lunch. He is 50, and you find out that his main problem is low back pain. This started some months ago at work, but he's now taken early retirement. Since stopping work the pain has started to bother him a bit more, but he really wants to get out and do things. He's been taking some paracetamol most days and it's helped a little."

Proposed vignette responses (Tables 4 &5) constituted advice that either was or was not in line with the best available evidence. Participants were asked to select all those proposals that they might recommend in practice.

### Administration

Questionnaires were given to pharmacists attending continuing education workshops in England organised by the University of Manchester's Centre for Pharmacy Post-graduate Education (CPPE) in Autumn 2005. Local facilitators (95) were each provided with 20 copies of the questionnaire together with a letter explaining its purpose and asking them to distribute the questionnaires. Pharmacists who wished to participate did so before or after the main workshop sessions; completed questionnaires were collected and returned by workshop facilitators to the University of Leeds for analysis. The workshops were varied and not chosen to involve the management of back pain.

CPPE is funded by the Department of Health (DH) to provide continuing education for registered pharmacists providing NHS services in England. Ethical approval was given by the University of Manchester's Committee on the Ethics of Research on Human Beings and the University of Leeds acted as sponsor under the terms of the DH Research Governance Framework [16].

### Data analysis

Data were entered into SPSS for Windows for processing and analysis. Analysis was restricted to community pharmacy respondents, as they were most likely to give OTC advice. CPPE workshops are also open to hospital and primary care pharmacists but they do not have direct contact with the general public and they responded in relatively small numbers. In order to check for representativeness, the age and sex profile of respondents was compared with the Royal Pharmaceutical Society of Great Britain's (RPSGB) register of pharmacists based in England [17], and their role compared with those reported in the RPSGB's 2003 census [11], using χ^2 ^tests. The responses to the vignettes that were in line with current clinical guidelines were agreed by three senior authors (JKM, GW and KB) and the most frequent responses were compared to this 'ideal'. The mean BBQ score was compared with other published values and appropriate statistical tests (such as *t*-tests and correlation coefficients) were used to explore the relationship between individual scores and potential explanatory variables. The relationship between attitude/vignette responses and experience of back pain/years of practice was examined using the χ^2 ^test of proportions.

## Results

### Response and demographics

Eighty facilitators returned a total of 402 completed questionnaires. In the completed questionnaires, 335 respondents stated that their main job was as a community pharmacist with some direct responsibility for OTC sales and advice. Comparison of the sample and RPSGB data (Table 1) shows significant differences. Middle aged pharmacists, women, pharmacy managers and locums were over-represented. In addition, 55% of the sample worked full time compared to 68% of pharmacists in general [11]. Regarding personal experience of back pain: 94 (28.2%) respondents reported none; 199 (59.8%) reported short term and 40 (12%) reported long-standing.

### Attitudes towards back pain and its treatment

The mean (SD) BBQ score for this sample of pharmacists was 31.37 (5.75). Univariate analysis showed no statistically significant relationship between BBQ score and age, gender or years since qualification. Those with no experience of back pain and those with long term back pain (n = 134, mean BBQ = 30.23) had lower scores than those with experience of short term back pain (n = 199, mean BBQ = 32.13) (p = 0.003). In total, 58.4% of the sample disagreed with the first WBS statement (advocating rest) in Table 2, and a slightly larger percentage (64.5%) agreed with the second WBS statement (advocating activity). Most (72%) disagreed with the idea of waiting until being pain free before returning to work and 86.5% supported the use of painkillers.

### Frequency and quality of back pain advice in the pharmacy

Pharmacists' perceptions of back pain advice in the pharmacy and their relationship to personal experience of back pain are shown in Table 3. There was a consistent (and plausible) relationship between responses to these questions and back pain. Those with more personal experience of back pain seem to demonstrate more confidence (fewer negative feelings, more advice opportunities and better advice provision) in their self perception of advice given in the pharmacy. In response to a question about *The Back Book*, 263 respondents said they had never heard of it and only 29 (9%) said they recommended it.

### Clinical case studies

In Vignette A, the most frequent response to each proposal was evidence based in most cases (Table 4). The exception was reassurance that back pain affects most people and settles quickly, which most respondents would not give. Respondents who would offer this valid reassurance had more post-qualification work experience then those that did not (25.8 vs. 21.3 years p = 0.000). Respondents giving the most frequent response to proposals 1 (p = 0.002), 4 (p = 0.004) and 5 (p = 0.034) also had more work experience than those that did not. Personal experience of back pain had a significant impact on response for 4 out of 7 proposals. In three of these proposals (1, 3 and 4) movement was viewed more positively by those with experience of back pain.

In Vignette B, the most frequent response to each proposal was evidence based in all cases (Table 5). However, around one-third of respondents would give advice leading to the restriction of movement or normal activities. There were no significant relationships between years of post-qualification work experience and responses to Vignette B. A significant relationship between experience of back pain and advice about movement was shown for Vignette B's proposal 4, which was selected by 55.6% of those with no experience of pain, 70.6% of those with short term experience and 83.8% of those with long term experience (χ^2 ^= 11.2, df = 2, p = 0.004).

### Education and training needs

When asked about the benefits of education and/or training concerning back pain, 93.1% (297/319) of respondents agreed that they would benefit personally and 93.5% (289/309) agreed that their staff would benefit.

## Discussion

This survey represents a first attempt to understand the nature and variety of advice that patients with back pain in England are likely to receive in the community pharmacy setting. Due to the method of distribution, the sample was not totally representative of community pharmacists in general. It contained more middle aged, female and part-time workers than expected. Some facilitators chose not to distribute the questionnaire and the total number of pharmacists given the opportunity to complete it is unknown. The method of distribution, which was judged to be the most efficient way to obtain a sufficiently large and diverse sample, made it impossible to know what the response rate was. The responses obtained are likely to represent pharmacists in England who were more motivated and interested in the topic. It is noteworthy that even in this self-selected group somewhat maladaptive beliefs were often reported and many did not feel confident to provide the best advice to customers with back pain.

About 72% of the respondents had some personal experience of back pain, which was a relatively high percentage given their age profile and the known epidemiology. It may be that people who have suffered from back pain were more likely to respond. However, the respondents' experience of back pain was not inconsistent with published data from international studies reporting that 59–84% suffer back pain at some stage in their lives [18].

The mean BBQ score (31.37) was slightly more positive than that found in similar surveys of lay people. At baseline in the UK BEAM trial the mean BBQ scores in the randomised groups (patients with low back pain) were about 28, and the highest score achieved by any group post-intervention was 31.3 [19]. Following a population based public health intervention in Australia (designed to alter attitudes towards back pain) the highest BBQ score achieved was 29.7 [13]. Pharmacists' responses to the WBS questions (approximately 60% agreement with activity statements) were similar to those in the Scottish general public after a public health campaign [9]. The BBQ has not previously been used in studies of healthcare practitioners. It was originally designed for use with people in industrial settings rather than patients.

The positive attitudes identified in this survey may be due to the nature of the sample, or a general change over time. Pharmacists who have had (and perhaps recovered from) short term back pain were more positive than the sample in general. The respondents may have been more knowledgeable and positive than the average pharmacist, because they were (a) attending a continuing education event in their own time and (b) motivated enough to respond to this survey. Unfortunately, because of the method of distribution there was no information about education session attendees and no further way to assess response bias.

Finding a more positive attitude among sufferers of back pain contrasts with the findings from a survey of public perceptions about back pain carried out in the UK ten years ago [20]. Those who had experienced back pain were less positive and especially those who had consulted a GP had more cautious or negative attitudes. Furthermore, in this public perceptions survey, those who were in older age groups and who were in social classes A-C were slightly more likely to agree with positive statements from the Back Book. The responding pharmacists in this present study would largely belong to these categories.

There seemed to be majority support for an active management approach to back pain: positive attitude towards back pain, positive attitude towards working when not pain free and agreement about using painkillers. Pharmacists' attitudes about working when not pain free were similar to those found in Australian and UK studies of GPs [13,14]. Promoting greater awareness of *The Back Book *among pharmacists is one obvious way to make evidence based information directly available to their staff and customers [15,21].

Vignettes are reported to be a valid way to collect information about the quality of clinical practice when compared with standardised patients (the gold standard method) [22]. However, actual practice has not been assessed in this survey, only possible practice based on existing knowledge. Personal experience of back pain seemed to influence the advice pharmacists say they would give, which has implications for the quality and consistency of advice given. The impact of attitudes on practice has previously been demonstrated in physiotherapists [4] and family physicians [5].

## Conclusion

The survey showed that pharmacists were willing and able to provide evidence based advice to people with low back pain. However, the representativeness of the results was unclear and further research would be useful. Those sampled were generally positive in their approach to the condition and, importantly, unlikely to recommend rest. Yet, in contrast, they also exhibited caution regarding movement that may cause pain, which could cause some confusion and lead to undue restriction of activity. Similar issues have been identified for family physicians and physical therapists [7]. Most pharmacists agreed that they would benefit from more training and education about back pain. Training to improve the confidence and ability of pharmacists to provide advice about the management of back pain may provide a useful model for improving other aspects of supported self-care, especially for conditions involving some chronic pain. This would support UK Government policy for making support for self-care in long term conditions widely available via pharmacies [23].

## Competing interests

The author(s) declare that they have no competing interests.

## Authors' contributions

JS, JKM and HE conceived and developed the study. JS conducted the data analysis. JKM, GW and AKB provided advice on the evidence based treatment of back pain. GW and AKB acted as expert consultants at all stages of study design and data analysis. JS prepared the draft manuscript. All the authors contributed to, reviewed and approved the final manuscript.

## Pre-publication history

The pre-publication history for this paper can be accessed here:



## Figures and Tables

**Table 1:** Age, sex and role of community pharmacist respondents

	*This survey*		*Register England 2004 [17]*		*χ^2 ^test of proportions*
		
	*Number*	*Percentage*	*Number*	*Percentage*	
*Age Group*					
29 years and under	27	8.1	6855	19.6	*χ^2 ^= 108.7*
30 to 39 years	69	20.6	9093	26.0	*p *< 0.01
40 to 49 years	104	31.0	7869	22.5	
50 to 59 years	92	27.5	5001	14.3	
60 to 64 years	29	8.7	1609	4.6	
65 years and over	14	4.2	4546	13.0	
Total	335	100	46241	100	
*Sex*					
Male	120	35.9	21484	46.5	*χ^2 ^= 14.8*
Female	214	64.1	24757	53.5	*p *< 0.01
Total	334	100	46241	100	
*Role*			*RPSGB Census 2003 [11]*		
Pharmacy owner	35	10.4	3076	16.5	*χ ^2 ^= 52.3*
Pharmacy manager	122	36.4	5214	28.0	*p *< 0.01
Locum	153	45.7	6562	35.3	
Other employee*	25	7.5	3750	20.2	
Total	335	100	18602	100	

*Second pharmacist in store or relief pharmacist for group of stores.

**Table 2:** Attitudes towards back pain and its treatment

*Statements (n = 333 unless stated)*	*Number (%) of Respondents*				
	
	*Strongly disagree*				*Strongly agree*
If you have back pain you should rest until it gets better (n = 332) (WBS)	75 (22.6)	119 (35.8)	82 (24.7)	50 (15.1)	6 (1.8)
If you have back pain you should try and stay active (WBS)	2 (0.6)	23 (6.9)	93 (27.9)	130 (39.0)	85 (25.5)
If you have back pain you should not return to work until free of pain*	86 (25.8)	154 (46.2)	67 (20.1)	22 (6.6)	4 (1.2)
If you have back pain you can use painkillers to control the pain (new)	2 (0.6)	3 (0.9)	40 (12.0)	167 (50.2)	121 (36.3)

*Similar to statement used in Australia survey by Buchbinder [13], but word 'almost' deleted from original and reworded to match style of WBS.

**Table 3:** Back pain advice and pharmacists' experience of back pain

		*Number (%)*	
		
*Statement*	Pain*	*Agree*	*Disagree*
I often have negative feelings about advising people with back pain^+^	None	20 (21.5)	73 (78.5)
(n = 328) χ^2 ^= 6.4 df = 2 p = 0.040	Short	27 (13.8)	168 (86.2)
	Long	2 (5.0)	38 (95.0)
	Total	49 (14.9)	279 (85.1)
I most frequently refer people with back pain to their GP	None	31 (34.1)	60 (65.9)
(n = 321) χ^2 ^= 4.7 df = 2 p = 0.094	Short	46 (24.1)	145 (75.9)
	Long	7 (17.9)	32 (82.1)
	Total	84 (26.2)	237 (73.8)
Back pain is an important topic where I work	None	23 (25.3)	68 (74.7)
(n = 319) χ^2 ^= 17.8 df = 2 p = 0.000	Short	68 (36.0)	121 (64.0)
	Long	25 (64.1)	14 (35.9)
	Total	116 (36.4)	203 (63.6)
Where I work people often ask for advice about back pain	None	41 (45.6)	49 (54.4)
(n = 323) χ^2 ^= 8.3 df = 2 p = 0.015	Short	98 (50.8)	95 (49.2)
	Long	29 (72.5)	11 (27.5)
	Total	168 (52.0)	155 (48.0)
I offer good advice about back pain	None	66 (79.5)	17 (20.5)
(n = 304) χ^2 ^= 5.6 df = 2 p = 0.060	Short	163 (89.1)	20 (10.9)
	Long	35 (92.1)	3 (7.9)
	Total	264 (86.8)	40 (13.2)
My support staff offer good advice about back pain	None	55 (67.1)	27 (32.9)
(n = 298) χ^2 ^= 4.2 df = 2 p = 0.122	Short	138 (77.1)	41 (22.9)
	Long	24 (64.9)	13 (35.1)
	Total	217 (72.8)	81 (27.2)

*Personal experience of back pain: none; short term or long-standing.

^+^Similar to Buchbinder [13]: 'advising people with back pain' replaces 'treating these people'.

**Table 4:** Responses to Vignette A and their association with experience of pain

		*Number (%)*	
		
*Suggestions (n = 315)*	Pain*	*Yes*	*No*
1. Advise her that she must continue to rest?	None	24 (27.0)	65 (73.0)
χ^2 ^= 6.7 df = 2 p = 0.035	Short	34 (18.1)	154 (81.9)
	Long	3 (7.9)	35 (92.1)
	Total	61 (19.4)	254 (80.6)
2. Suggest adequate analgesia?	None	85 (95.5)	4 (4.5)
χ^2 ^= 2.4 df = 2 p = 0.300	Short	177 (94.1)	11 (5.9)
	Long	38 (100)	0 (0)
	Total	300 (95.2)	15 (4.8)
3. Tell her to avoid any movement that hurts?	None	45 (50.6)	44 (49.4)
χ^2 ^= 6.5 df = 2 p = 0.040	Short	85 (45.2)	103 (54.8)
	Long	10 (26.3)	28 (73.7)
	Total	140 (44.4)	175 (55.6)
4. Suggest that swimming or movement in water might be helpful?	None	59 (66.3)	30 (33.7)
χ^2 ^= 9.5 df = 2 p = 0.008	Short	142 (75.5)	46 (24.5)
	Long	35 (92.1)	3 (7.9)
	Total	236 (74.9)	79 (25.1)
5. Suggest that carrying on with her usual activities as far as possible is the best thing she could do?	None	43 (48.3)	46 (51.7)
χ^2 ^= 0.7 df = 2 p = 0.701	Short	99 (52.9)	88 (47.1)
	Long	21 (55.3)	17 (44.7)
	Total	163 (51.9)	151 (48.1)
6. Reassure her that back pain affects nearly everyone at some time but usually settles quickly?	None	30 (33.7)	59 (66.3)
χ^2 ^= 11.6 df = 2 p = 0.003	Short	102 (54.3)	86 (45.7)
	Long	22 (57.9)	16 (42.1)
	Total	154 (48.9)	161 (51.1)
7. Warn her that back pain often gets worse before it gets better?	None	7 (7.9)	82 (92.1)
χ^2 ^= 1.2 df = 2 p = 0.546	Short	11 (5.9)	177 (94.1)
	Long	4 (10.5)	34 (89.5)
	Total	22 (7.0)	293 (93.0)

*See first footnote to Table 3.

**Table 5:** General response to Vignette B

	*Number (%)*	
	
*Suggestions (n = 315)*	*Yes*	*No*
1. Suggest adequate analgesia?	255 (81.0)	60 (19.0)
2. Advise him that he should be careful and avoid doing anything that is painful?	97 (30.8)	218 (69.2)
3. Ask him if he has tried either a cold pack or applying local heat for short term pain relief?	198 (62.9)	117 (37.1)
4. Suggest that movement is good for back pain and regular walks might help?	215 (68.7)	100 (31.7)
5. Warn him that going back to work would be likely to make his back worse?	13 (4.1)	301 (95.9)
6. Suggest that he should try to keep doing his leisure activities?	192 (61.0)	123 (39.0)
7. Suggest that since he still has quite a lot of pain and is finding it difficult to get back to normal activities he might want to consult his GP or a therapist.	280 (88.9)	35 (11.1)

## References

[B1] Waddell G (2004). The Back Pain Revolution.

[B2] Airaksinen O, Brox JI, Cedraschi C, Hildebrandt J, Klaber Moffett J, Kovacs F, Mannion AF, Reis S, Staal JB, Ursin H, Zanoli G, On behalf of the COST B13 Working Group on Guidelines for Chronic Low Back Pain (2006). European guidelines for the management of chronic non-specific low back pain. European Spine Journal.

[B3] van Tulder M, Becker A, Bekkering T, Breen A, Gil del Real MT, Hutchinson A, Koes B, Laerum E, Malmivaara A, On behalf of the COST B13 Working Group on Guidelines for the Management of Acute Low Back Pain in Primary Care (2006). European guidelines for the management of acute non-specific low back pain in primary care. European Spine Journal.

[B4] Houben RMA, Ostelo RWJG, Vlaeyen JWS, Wolters PMJC, Peters M, Stomp-van den Berg SGM (2005). Health care providers' orientations towards common low back pain predict perceived harmfulness of physical activities and recommendations regarding return to normal activity. European Journal of Pain.

[B5] Rainville J, Carlson N, Polatin P, Gatchel RJ, Indahl A (2000). Exploration of physicians' recommendations for activities in chronic low back pain. Spine.

[B6] Pincus T, Foster NE, Vogel S, Santos R, Breen A, Underwood M Attitudes to back pain amongst musculoskeletal practitioners: a comparison of professional groups and practice settings using the ABS-mp. Manual Therapy.

[B7] Linton SJ, Vlaeyen J, Ostelo R (2002). The back pain beliefs of health care providers: are we fear-avoidant?. Journal of Occupational Rehabilitation.

[B8] Buchbinder R, Jolley D (2004). Population based intervention to change back pain beliefs: three year follow up population survey. BMJ.

[B9] Working Backs Scotland: Frequently asked questions. http://www.workingbacksscotland.scot.nhs.uk/faqs.htm#q4.

[B10] Davies J (2004). Community pharmacists to step up public health duties. Health Service Journal.

[B11] Hassell K (2004). An overview of the main findings of the 2003 pharmacy workforce census. Pharmaceutical Journal.

[B12] Symonds TL, Burton AK, Tillotson KM, Main CJ (1996). Do attitudes and beliefs influence work loss due to low back trouble?. Occup Med.

[B13] Buchbinder R, Jolley D, Wyatt M (2001). Population based intervention to change back pain beliefs and disability: three part evaluation. BMJ.

[B14] Chaudhary N, Longworth S, Sell PJ (2004). Management of mechanical low back pain – a survey of beliefs and attitudes in GPs from Leicester and Nottingham. Eur J Gen Prac.

[B15] Roland M (2002). The Back Book.

[B16] Department of Health (2005). Research Governance Framework for Health and Social Care, London.

[B17] Hassell K (2004). RPSGB Register Analysis 2004.

[B18] Walker BF (2000). The prevalence of low back pain: a systematic review of the literature from 1966 to 1998. J Spinal Disord.

[B19] UK BEAM Trial Team (2004). United Kingdom back pain exercise and manipulation (UK BEAM) randomised trial: effectiveness of physical treatments for back pain in primary care. BMJ.

[B20] Klaber Moffett J, Newbronner E, Waddell G, Croucher K, Spear S (2000). Public perceptions about low back pain and its management: a gap between expectations and reality. Health Expectations.

[B21] Burton AK, Waddell G, Tillotson KM, Summerton N (1999). Information and advice to patients with back pain can have a positive effect: a randomised controlled trial of a novel educational booklet in primary care. Spine.

[B22] Peabody JW, Luck J, Glassman P, Jain S, Hansen J, Spell M, Lee M (2004). Measuring the quality of physician practice by using clinical vignettes: a prospective validation study. Ann Intern Med.

[B23] Department of Health (2005). Choosing health through pharmacy: a programme for pharmaceutical public health 2005–2015, London.

